# Private Equity Acquisition of Gastroenterology Practices and Colonoscopy Price and Quality

**DOI:** 10.1001/jamahealthforum.2025.1476

**Published:** 2025-06-20

**Authors:** Daniel R. Arnold, Brent D. Fulton, Ola A. Abdelhadi, Arjun Teotia, Richard M. Scheffler

**Affiliations:** 1 Department of Health Services, Policy & Practice, School of Public Health, Brown University, Providence, Rhode Island; 2 School of Public Health, University of California, Berkeley; 3 Stanford University, Stanford, California; 4 Henry Ford Health, Detroit, Michigan; 5 Goldman School of Public Policy, University of California, Berkeley

## Abstract

**Importance:**

Private equity (PE) has rapidly been acquiring physician practices in the US, but a full understanding of its association with health care prices, spending, utilization, and quality is still unknown.

**Objective:**

To examine changes in colonoscopy prices, spending, utilization, and quality associated with PE acquisition of gastroenterology practices.

**Design, Setting, and Participants:**

This difference-in-differences event study and economic evaluation analyzed data from US gastroenterology practices that were acquired by PE firms between 2015 and 2021. Commercial claims covering more than 50 million enrollees were used to calculate price, spending, utilization, and quality measures from 2012 to 2021. The data were analyzed between April 2024 and September 2024.

**Exposures:**

PE acquisition of gastroenterology practices.

**Main Outcomes and Measures:**

The main outcomes were price, spending per physician, number of colonoscopies per physician, number of unique patients per physician, and quality (polypectomy detection, incomplete colonoscopies, and 4 adverse event measures: cardiovascular, serious gastroenterology, nonserious gastroenterology, and any adverse event).

**Results:**

Data from more than 1.1 million patients (mean [SD] age, 47.1 [8.4] years; 47.8% male patients) undergoing 1.3 million colonoscopies were analyzed. The sample included 718 851 treated colonoscopies conducted by 1494 physicians among 590 900 patients across 1240 PE-acquired practice sites and 637 990 control colonoscopies conducted by 2550 physicians among 527 380 patients across 2657 independent practice sites. Colonoscopy prices at PE-acquired gastroenterology practices increased by 4.5% (95% CI, 2.5%-6.6%; *P* < .001) relative to independent gastroenterology practices. The estimated price effect increased to 6.7% (95% CI, 4.2%-9.3%; *P* < .001) when considering only colonoscopies performed by gastroenterologists in PE-acquired practices with market shares above the 75th percentile (24.4%) in 2021 as treated. Colonoscopy spending per physician increased by 16.0% (95% CI, 8.4%-24.0%; *P* < .001), while the number of colonoscopies and the number of unique patients per physician increased by 12.1% (95% CI, 5.3%-19.4%; *P* < .001) and 11.3% (95% CI, 4.4%-18.5%; *P* < .001), respectively; however, these spending and utilization measures were already increasing prior to PE acquisition. No statistically significant associations were detected for the 6 quality measures analyzed.

**Conclusions and Relevance:**

In this economic evaluation, PE acquisition of gastroenterology practices led to higher prices and spending but had no discernible effect on quality. Policymakers may be well advised to monitor PE investment in physician practices given the increase in prices and spending without a commensurate increase in quality.

## Key Points

**Question:**

What is the association between private equity (PE) acquisition and commercial prices, spending, utilization, and quality at gastroenterology practice sites?

**Findings:**

This economic evaluation of more than 1.1 million patients undergoing 1.3 million colonoscopies found that colonoscopy prices, spending, and utilization increased at PE-acquired gastroenterology practices, but PE acquisition was not associated with changes in quality.

**Meaning:**

Without commensurate quality improvements, it is difficult to justify acquisitions associated with higher prices, spending, and utilization.

## Teaser

This economic evaluation assesses how private equity ownership of gastroenterology practices affects colonoscopy prices, spending, utilization, and quality.

## Introduction

Physician practices in the US are rapidly being acquired by private equity (PE) firms. A recent study found a 6-fold increase in the number of PE acquisitions of physician practices between 2012 and 2021.^1^ These acquisitions are occurring in many specialties, with the highest share of physicians working in practices owned by PE firms occurring in gastroenterology (13.1%).^2^ This acquisition activity has also been reported in studies published in gastroenterology specialty journals.^3,4,5^ While the influx of capital from PE has the potential to create technological and operational efficiencies, many have feared that PE’s short-term financial incentives may negatively impact health care prices, quality, or access.

The literature on the impact of PE acquisitions on the prices and quality of physician practices is small but growing. We identified 10 studies that used quasi-experimental research designs to estimate the association between PE acquisitions (or investments) and physician practice outcomes, such as prices, spending, utilization, and quality.^1,6,7,8,9,10,11,12,13,14^ Several of these studies examined price, spending, and utilization, finding they were almost always positively associated with PE acquisitions across a range of specialties, including gastroenterology,^6,7^ anesthesiology,^11^ dermatology,^6^ ophthalmology,^6,13,14^ and neonatology.^9^

Previous studies often compare changes in the average allowed amount per claim for physicians (of a particular specialty) at practices before and after acquisition. The average allowed amount per claim would increase if the prices of all (or most) procedures increased, but would also increase if physicians simply shifted to performing more expensive procedures postacquisition. In this study, we are interested in isolating the former price effect. To do this, we focus on colonoscopies. We chose colonoscopies because of their importance, frequency, and presence in health care claims data. Colorectal cancer is the third leading cause of cancer death, but many deaths have been prevented because of early detection from screening, including the approximately 15 million colonoscopies performed each year in the US.^15,16^ The American College of Gastroenterology/American Society for Gastrointestinal Endoscopy Quality Task Force periodically publishes recommendations and quality measures to improve the technical performance of colonoscopies.^17^ This task force establishes performance targets for both outcome and process measures. The US Preventive Services Task Force currently recommends screenings to begin at 45 years of age, with a colonoscopy occurring every 10 years, tapering off to only selective screening for those aged 76 to 85 years.

This article makes two primary contributions to the literature. First, we estimate the association between PE acquisition and price and quality for the same sets of acquired and control gastroenterology physician practices. Previous studies on PE price and spending effects typically do not also assess associations with quality. We analyze the association between PE acquisition and 6 quality measures (polypectomy detection, incomplete colonoscopies, and 4 adverse event measures: cardiovascular, serious gastroenterology, nonserious gastroenterology, and any adverse event). Second, we explore how the PE price effect varies based on the gastroenterology practice’s market share.

## Methods

This economic evaluation was approved by the institutional review board of the University of California, Berkeley. Informed consent was waived because the study used only deidentified data. The study followed the Consolidated Health Economic Evaluation Reporting Standards (CHEERS) reporting guideline.

Constructing our analytic sample was a 3-step process. First, we identified PE acquisitions of physicians using data from PitchBook and Irving Levin Associates. Second, we linked this information to physician characteristics provided by IQVIA. Third, we calculated price, spending, utilization, and quality outcome variables from commercial claims data provided by the Health Care Cost Institute (HCCI) and merged these measures onto our physician characteristics file. After our analytic file was constructed, we estimated event study models for each of our outcomes of interest.

### PE Acquisitions and Physician Practice Characteristics

We identified gastroenterology practices acquired by PE firms from January 2012 to December 2021 using reported transactions in PitchBook and Irving Levin Associates Healthcare M&A Database. We then linked the compiled acquisition data to 2 IQVIA physician datasets: the OneKey Database provided by IQVIA for 2020 to 2021 and the SK&A Office-Based Physicians Database provided by IMS Health (now IQVIA) for 2012 to 2019, which have been used in prior studies.^1,6,18^ In 2021, the OneKey Database had information on 13 702 gastroenterologists, or 87% of the active gastroenterologists in the US as reported by the Association of American Medical Colleges.^19^ SK&A also had good coverage of gastroenterologists throughout our study period, with information on 12 074 gastroenterologists (94% of active gastroenterologists at the time) in 2012.

To match physician practices to acquisition datasets, we used record-linking methods based on practice name and location. Once we identified a practice as acquired by PE, it was considered a PE-acquired practice for the duration of the study period unless it experienced a change in ownership to a non-PE entity. Additionally, we performed a manual search to add to our ability to identify as many practices owned by PE firms in the IQVIA data as possible. Our match rates for each year during the study period varied from 70% to 84%, with a weighted mean of 75%.

### Outcome Variables

All of our outcome variables were calculated from HCCI’s commercial claims database. The HCCI database contains commercial claims from Blue Cross Blue Shield, Aetna, and Humana covering more than 55 million enrollees, or roughly one-third of commercial enrollees. We used physician claims from 2012 to 2021, with our unit of analysis being a colonoscopy. The Healthcare Common Procedure Coding System codes we used to identify colonoscopies are shown in eTable 1 in Supplement 1. Physician national provider identifiers were used to link the physician characteristics dataset to claims. Claims were found for 87% of the gastroenterologist national provider identifiers in our physician characteristics dataset.

We analyzed 10 outcome variables: 1 price variable, 1 spending variable, 2 utilization variables, and 6 quality variables. Colonoscopy was the unit of analysis for price and the 6 quality measures. The unit of analysis for the 3 spending and utilization variables was physician-year. Price was measured as the allowed amount for the colonoscopy procedure, which includes reimbursement by insurers and any patient cost-sharing. The spending variable we analyzed was the total allowed amount for colonoscopies performed by a physician in a given year. The 2 utilization variables we analyzed were the number of colonoscopies per physician per year and the number of unique patients per physician per year.

We used a similar set of quality measures as a recent study that analyzed the impact of vertical integration on colonoscopy quality.^20^ Polypectomies and incomplete colonoscopies were selected for analysis because they have the potential to be affected by variations in gastroenterology practices, and they are obtainable from claims data. Studies have shown that adenoma detection rate (ADR) (ie, the proportion of patients with at least 1 colorectal adenoma detected during colonoscopy) is the most clinically relevant and best validated measure for how a colonoscopy is performed.^17,21^ Because measuring ADR from claims is difficult, we instead use the polypectomy rate, given that it has been shown to correlate well with ADR.^22,23^ Gastroenterology physicians with higher polypectomy rates generally have better patient outcomes, such as lower interval cancer.^24,25^ We identified polypectomy rates from Healthcare Common Procedure Coding System codes on concurrent pathology bills. Incomplete colonoscopies are an indicator of poor quality because they can result in missed lesions, a contributor to interval cancer.^26^

The next set of quality indicators was adverse events.^17^ The 4 postprocedure complication quality indicators we analyzed included 3 binary indicators (cardiovascular complications, serious gastroenterology complications, and nonserious gastroenterology complications) with the fourth measure indicating whether any of these complications occurred. Cardiovascular complications included arrhythmia, congestive heart failure, cardiac or respiratory arrest, syncope, hypotension, and shock. Serious gastroenterology complications included perforation, bleeding, or infection. Perforation of the colon after colonoscopy is a significant adverse outcome that can ultimately result in death. Large studies put the rate of perforation at between 0.005% to 0.085%, so it is a rare complication. Bleeding and infection are more common complications after colonoscopy. We searched for *International Classification of Diseases, Ninth Revision (ICD-9)*/*ICD-10* codes indicating cardiovascular gastroenterology complications in the claims (inpatient, outpatient, physician) of the patient receiving the colonoscopy up to 30 days after the colonoscopy. We identified perforation and infection events using *ICD-9/ICD-10* codes that are present either on or within 7 days after colonoscopy. For bleeding events, we searched for the presence of *ICD-9/ICD-10* codes up to 7 days after the colonoscopy date, as bleeding might also be a reason for a visit rather than the outcome.

Nonserious gastroenterology complications included paralytic ileus, nausea, vomiting, dehydration, abdominal pain, diverticulitis, and enterocolitis. We searched for *ICD-9/ICD-10* codes indicating nonserious gastroenterology complications on claims up to 30 days after the colonoscopy. The *ICD-9/ICD-10* codes we used to calculate all 6 of our quality measures are shown in eTable 1 of Supplement 1. Our final quality measure (any complication) equaled 1 if the patient experienced any cardiovascular, serious gastroenterology, or nonserious gastroenterology complications.

### Statistical Analysis

Our empirical strategy is based on a difference-in-differences event study with physician and year fixed effects. The unit of analysis in our model is a colonoscopy, and we use various characteristics of patients, physicians, and markets as controls. The treatment variable in our setting is based on the PE ownership variable of the physician who performs the procedure. Only physicians in practices acquired by PE firms from 2015 to 2021 were considered treated so that all treated physicians had a preperiod of at least 3 years (ie, 2012-2014). The comparison group of physicians included only physicians who were independent throughout the study period, with independent being defined as employed by a practice that was not owned by a corporate entity (eg, a health system).

In our event study analyses, event time 0 denotes the year the physician’s practice was acquired by PE. We used data from 6 years before to 6 years after acquisition, with the year prior to acquisition as the reference period. Our event study model compared changes in outcomes of interest in PE-acquired practices with independent practices, before and after acquisition. In the context of heterogeneous treatment effects and staggered interventions, which is the context of the present study, recent econometric literature has shown that the coefficients arising from ordinary least-squares 2-way fixed-effect events can be biased.^27,28^ To avoid this bias, all the event studies we present are based on the Sun and Abraham^29^ model that produces unbiased event study estimates (eMethods in Supplement 1). Significance testing was 2-sided, and *P* < .05 was considered statistically significant. Statistical analyses were performed using Stata 18.0 (StataCorp). The data were analyzed between April 2024 and September 2024.

## Results

Data from 1.1 million patients (mean [SD] age, 47.1 [8.4] years; 47.8% male patients) undergoing 1.3 million colonoscopies at 3897 practice sites were analyzed. Table 1 shows descriptive statistics for the 718 851 treated colonoscopies (590 900 patients) and 637 990 control colonoscopies (527 380 patients) in the study sample. The treated colonoscopies were performed by 1494 physicians across 1240 PE-acquired practice sites. Between 80 and 351 gastroenterologists were employed across 104 to 354 practice sites acquired by PE in each year from 2015 to 2021 (eTable 2 in Supplement 1). The control colonoscopies were performed by 2550 physicians across 2657 independent practice sites (see eTable 3 in Supplement 1 for the number of treated and control physicians and practice sites in the sample by year and eTable 4 in Supplement 1 for the number of years that each practice contributes to the analytic sample). The average colonoscopy price was $423 at PE-acquired practices and $425 at independent practices. Patients at PE-acquired practices were more likely to have incomplete colonoscopies and have a postprocedure infection, but were less likely to bleed or have a serious gastroenterology complication. Across most characteristics, the differences between PE-acquired and control colonoscopies are small in magnitude. eFigure 1 in Supplement 1 shows raw trends for price, spending, utilization, and patients per physician for both treated and control physicians.

**Table 1.** Outcome Variables and Patient Characteristics by Treatment Status^a^

Measure	Mean (SD)		*P* value for difference in means
	Treated (PE-acquired practices)	Control (independent practices)	
Sample sizes, No.			
Colonoscopies	718 851	637 990	NA
Patients	590 900	527 380	
Physicians	1494	2550	
Practice sites	1240	2657	
Price, utilization, and spending measures			
Price, $	423.26 (191.93)	424.54 (241.47)	.001
Colonoscopies per physician-year, No.	58.58 (61.01)	41.85 (58.56)	<.001
Patients undergoing colonoscopy per physician-year, No.	48.73 (51.12)	34.87 (48.32)	<.001
Colonoscopy spending per physician-year, $	24 790.65 (31 479.65)	17 771.06 (31 025.47)	<.001
Process measures			
Polypectomy	0.63 (0.48)	0.62 (0.48)	<.001
Incomplete colonoscopy	0.01 (0.07)	0.004 (0.06)	<.001
Postprocedure complication measures			
Cardiovascular	0.01 (0.08)	0.01 (0.08)	.16
Serious gastroenterology	0.01 (0.11)	0.02 (0.13)	<.001
Nonserious gastroenterology	0.02 (0.15)	0.03 (0.16)	<.001
Any complication	0.04 (0.20)	0.046 (0.21)	<.001
Patient characteristics			
Age, y			
0-17	0.001 (0.03)	0.003 (0.06)	<.001
18-24	0.02 (0.13)	0.02 (0.14)	
25-34	0.04 (0.20)	0.04 (0.20)	
35-44	0.09 (0.28)	0.09 (0.29)	
45-54	0.38 (0.48)	0.37 (0.48)	
55-64	0.47 (0.50)	0.48 (0.50)	
Sex, No. (%)			
Female	378 403 (52.6)	330 734 (51.8)	<.001
Male	340 448 (47.4)	307 256 (48.2)	

Abbreviations: NA, not applicable; PE, private equity.

^a^ Data sources included Health Care Cost Institute, PitchBook, Irving Levin Associates, and IQVIA’s OneKey Database.

**Figure 1.**
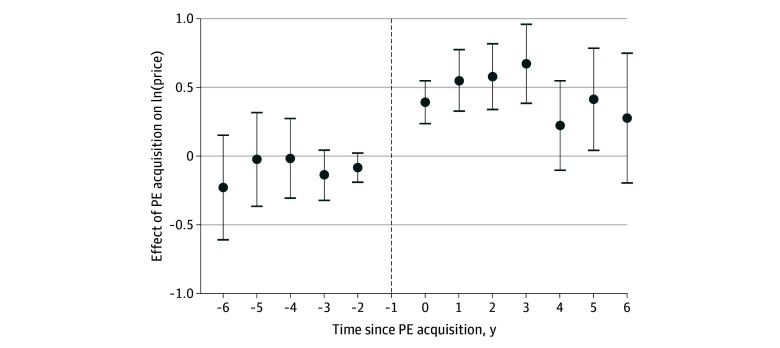
Price Event Study The figure uses Sun and Abraham^29^ event study coefficients. The unit of analysis was a colonoscopy. Each regression included physician and year fixed effects as well as controls for patient age (measured in age bands), sex, and *Current Procedural Terminology* code. Standard errors are clustered at the practice site level. The dashed vertical line represents the year prior to acquisition, which was considered the reference period in this study. The error bars represent the 95% CIs for each estimate. PE indicates private equity.

Figure 1 graphically depicts the results of the price analysis. The preperiod coefficient estimates (from 6 to 2 years before) all hover around zero and are not statistically significant. The coefficient estimates become positive and statistically significant immediately after PE acquisition. The 0.04 (95% CI, 0.02-0.06; *P* < .001) coefficient at event time 0 indicates prices were 4.0% higher relative to control practices in the year of the acquisition using the following equation: (exp[0.039] − 1) × 100. The estimated price effect reached 7.8% (95% CI, 4.9%-10.6%; *P* < .001) 3 years after acquisition.

**Figure 2.**
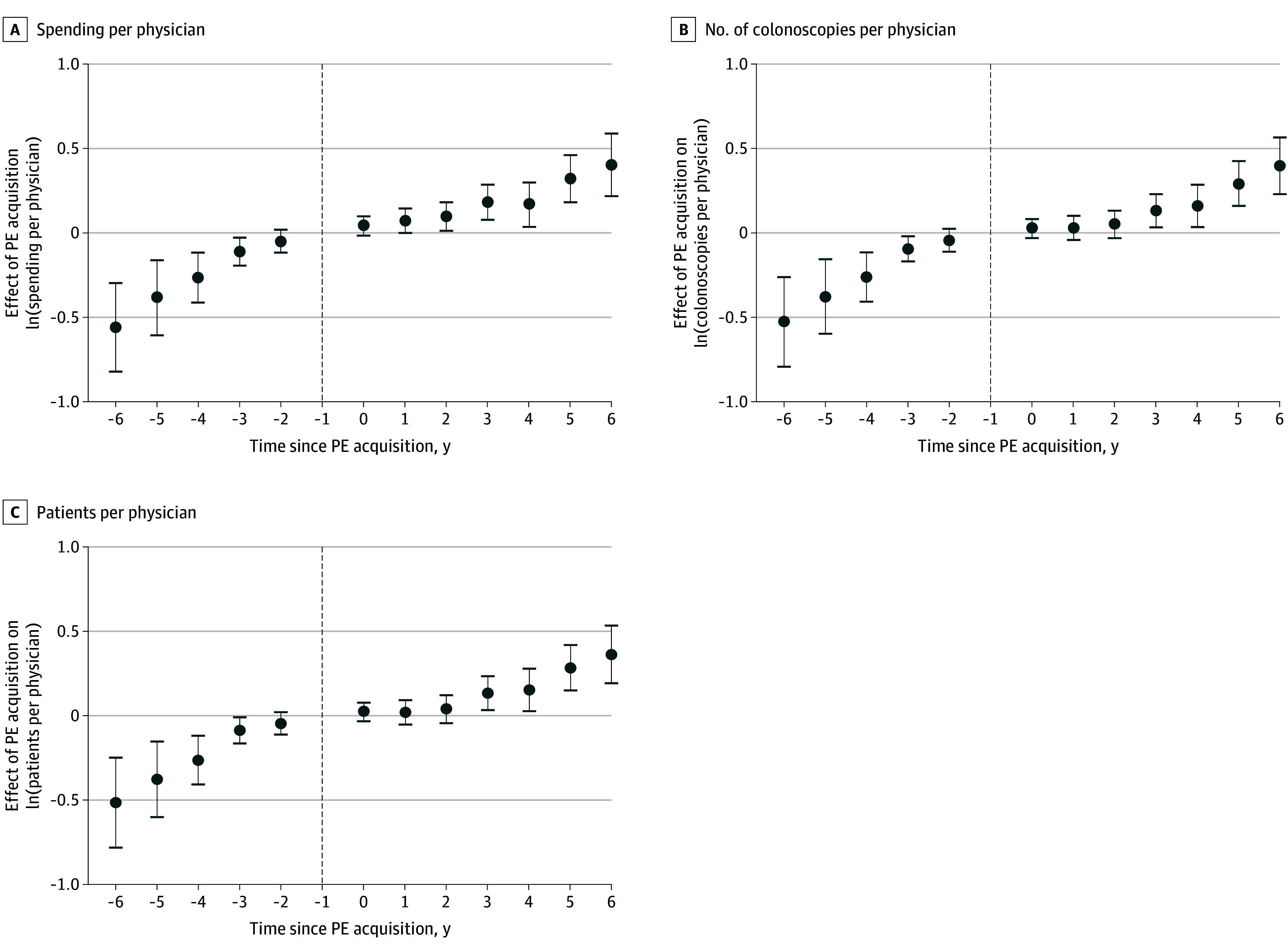
Spending and Utilization Event Studies The figure uses Sun and Abraham^29^ event study coefficients. The unit of analysis was a colonoscopy. Each regression included physician and year fixed effects as well as controls for patient age (measured in age bands), sex, and *Current Procedural Terminology* code. Standard errors are clustered at the practice site level. The dashed vertical line represents the year prior to acquisition, which was considered the reference period in this study. The error bars represent the 95% CIs for each estimate. PE indicates private equity.

Figure 2 shows the results of the spending and utilization analyses. For all 3 outcome variables, the gap between PE-acquired and control practices grows gradually from the year of acquisition all the way until 6 years postacquisition, the maximum number of years postacquisition observed in the data. At 6 years postacquisition, colonoscopy spending per physician, the number of colonoscopies per physician, and the number of patients per physician were 50.0% (95% CI, 24.7%-80.4%; *P* < .001), 48.8% (95% CI, 25.8%-76.2%; *P* < .001), and 43.8% (95% CI, 21.2%-70.7%; *P* < .001), respectively, above the corresponding measures for control practices. However, these results should be interpreted with caution, as it is clear from each of the graphs that these measures were increasing for the PE-acquired practices prior to acquisition.

**Figure 3.**
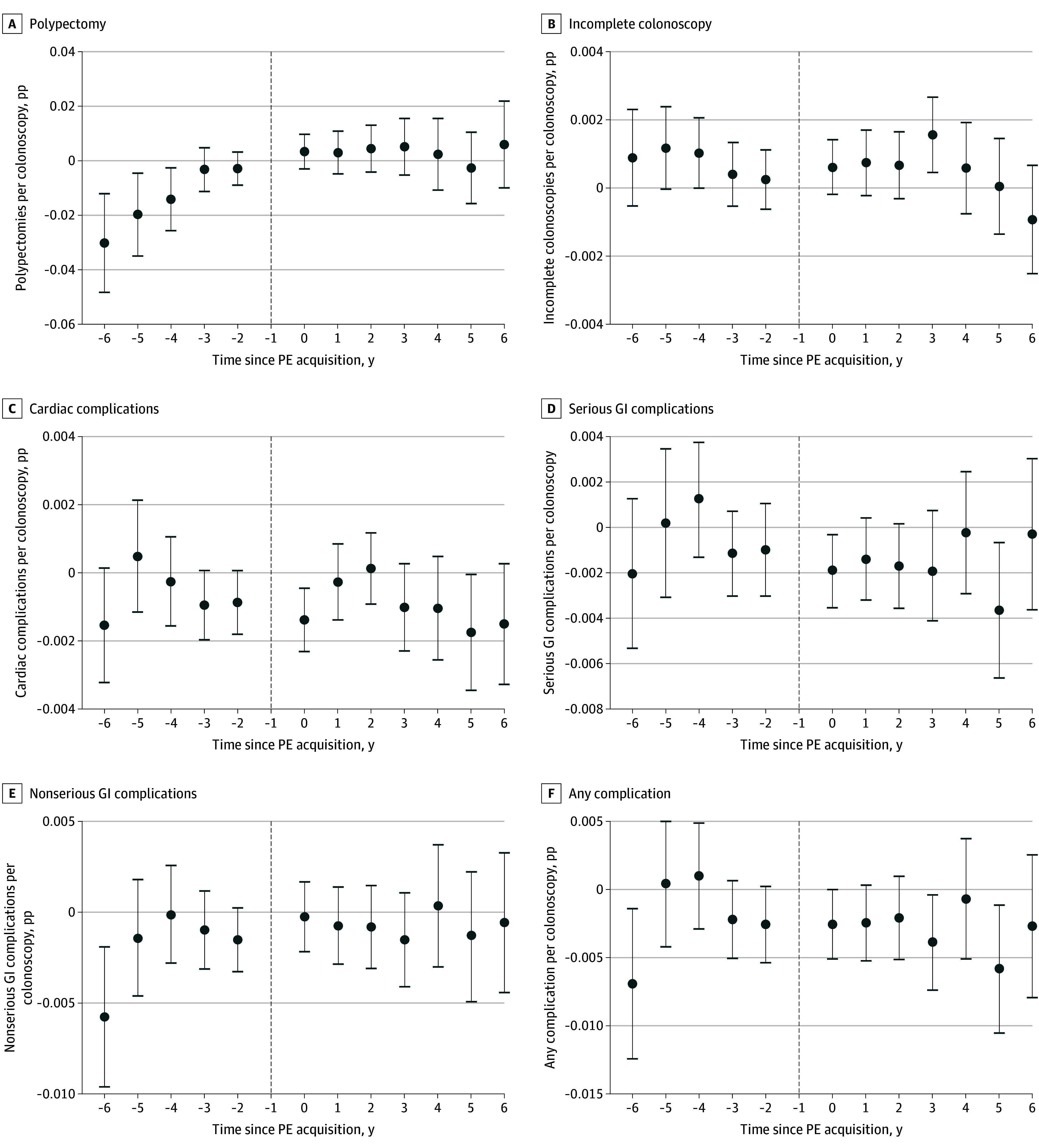
Quality Event Studies The figure uses Sun and Abraham^29^ event study coefficients. The unit of analysis was a colonoscopy. Each dependent variable is a 0/1 variable. All regressions included physician and year fixed effects as well as controls for patient age (measured in age bands), sex, and *Current Procedural Terminology* code. Standard errors are clustered at the practice site level. The dashed vertical line represents the year prior to acquisition, which was considered the reference period in this study. The error bars represent the 95% CIs for each estimate. Serious gastroenterology (GI) complications included perforation, bleeding, or infection. PE indicates private equity; pp, percentage points.

Figure 3 presents the results of the quality analyses. There was no effect of PE acquisition on quality for any of the 6 quality measures analyzed.

Table 2 summarizes the results of the previous 3 figures by showing the Sun and Abraham^29^ difference-in-differences coefficients as opposed to event study coefficients. The table shows prices, spending per physician, colonoscopies per physician, and patients undergoing colonoscopy per physician to be 4.5% (95% CI, 2.5%-6.6%; *P* < .001), 16.0% (95% CI, 8.4%-24.0%; *P* < .001), 12.1% (95% CI, 5.3%-19.4%; *P* < .001), and 11.3% (95% CI, 4.4%-18.5%; *P* < .001), respectively, higher than the corresponding measures for control practices.

**Table 2.** Summary of Difference-in-Differences Estimates^a^

Variable	Difference-in-differences estimate (95% CI)	*P* value
Price, utilization, and spending measures, %		
ln(Price)	4.5 (2.5 to 6.6)^a^	<.001
ln(Colonoscopy spending per physician)	16.0 (8.4 to 24.0)^a^	<.001
ln(Colonoscopies per physician)	12.1 (5.3 to 19.4)^a^	<.001
ln(Patients undergoing colonoscopy per physician)	11.3 (4.4 to 18.5)^a^	<.001
Quality measures		
Polypectomy	0.00013 (−0.00004 to 0.00029)	.13
Incomplete colonoscopy	0.00037 (−0.00024 to 0.00098)	.23
Cardiovascular complications	0.00060 (−0.00060 to 0.00180)	.33
Serious gastroenterology complications	−0.00127 (−0.00392 to 0.00137)	.35
Nonserious gastroenterology complications	0.00019 (−0.00271 to 0.00310)	.90
Any complication	−0.000650 (−0.00267 to 0.00137)	.53

^a^ The estimated difference-in-differences coefficients associated with the natural log dependent variables were converted to the percentages shown in the table using the formula (exp[coefficient] − 1) × 100. The unit of analysis was a colonoscopy. Each process and postprocedure complication measure is a 0/1 variable. All regressions included physician and year fixed effects as well as controls for patient age (measured in age bands), sex, and *Current Procedural Terminology* code. Standard errors are clustered at the practice site level.

The magnitude of the price effect was determined for the PE-acquired practices that had the largest metropolitan statistical area (MSA) market shares by the end of the study period in 2021. By 2021, the mean market share associated with treated colonoscopies was 14.9%, while the 25th percentile was 4.9%, and the median and 75th percentile were 10.5% and 24.4%, respectively.

Analyses were also conducted to determine whether colonoscopies performed by gastroenterologists in PE-acquired practices with market shares above the 75th percentile were associated with larger price increases than the average price increases reported by the main analyses. The control group for this analysis was the same as in the main results, except that gastroenterologists located outside of MSAs (2% of control gastroenterologists) were excluded. The estimated price effect increased to 6.7% (95% CI, 4.2%-9.3%; *P* < .001) (based on the estimate from a difference-in-differences version of the model) when considering only colonoscopies performed by gastroenterologists in practices with market shares above the 75th percentile (24.4%) in 2021. While the 6.7% estimate is larger than the point estimate of the main price effect, the 95% CIs are wide enough on the estimates that it cannot be determined whether the estimates are statistically different from each other. The event study for this analysis is presented as eFigure 2 in Supplement 1.

Two sensitivity analyses were conducted. First, the models were re-estimated using only data from 2012 to 2019 to rule out COVID-19 as having potentially influenced the results. COVID-19 shutdown procedures were staggered in different states that differed in their baseline penetration of PE. In addition, private practices with more autonomy over their procedures may have responded differently to elective procedures than hospitals or academic medical centers during this time. The overall takeaways from the main analysis, higher prices, spending, and utilization with no effect on quality, are the same for the 2012 to 2019 subsample. eTable 5 in Supplement 1 reproduces Table 2, but for the 2012 to 2019 study period. Second, models were re-estimated after excluding 2015 acquisitions. Larger PE acquisitions of gastroenterology practices took place in or after 2016. Excluding 2015 acquisitions increased the estimated price effect from 4.5% (95% CI, 2.5%-6.6%; *P* < .001) to 10.8% (95% CI, 8.1%-13.4%; *P* < .001). eFigure 3 in Supplement 1 shows the price event study graph after excluding 2015 acquisitions.

## Discussion

In this economic evaluation, we found that gastroenterology practices acquired by PE firms experienced a 4.5% price increase relative to controls, rising to 6.7% in MSAs where the practice had greater market power. Additionally, spending per physician increased by 16.0%, primarily driven by higher utilization. However, these increases in price and spending did not translate into improved quality of care for colonoscopies, one of the most common cancer screenings.

Evidence is mounting that corporate control in health care, including PE, has led to higher health care prices and spending without a commensurate increase in quality. Therefore, in spring 2024, the US Department of Health and Human Services, the Justice Department’s Antitrust Division, and the Federal Trade Commission asked the public for their input, particularly information about transactions that are not reportable to these agencies because they fall below the Hart-Scott-Rodino threshold ($126.4 million in 2025).^30^ Based on more than 2000 comments received, including many from physicians and patients, these agencies reported the public is concerned about corporate influence in health care, particularly PE firms, consistent with the evidence base of harmful effects.^31^ One theme that emerged was the need for increased transparency, including notification for proposed mergers and acquisitions in health care, which 15 states require, and for patients to be notified of changes in corporate ownership.

Although the prices increases we found were significant, they were generally less than the price increases found in other studies examining PE firm acquisitions of physician practices, which reported a broad range of increases across specialties: 0% to 5% in dermatology,^6,8^ 7.1% in ophthalmology,^6^ 26.0% in anesthesiology,^11^ 32.2% to 34.7% in gastroenterology,^6,7^ and 70.4% in neonatology.^9^ The higher price increases reported in other gastroenterology studies compared to our findings are likely due to differences in claims datasets and periods analyzed. Additionally, other studies often analyzed changes in the average allowed amount per claim, which would increase if physicians shifted to more expensive procedures. Our focus on one procedure (colonoscopies) avoids the procedure shifting possibility and is complementary to the approach taken by other studies, as the shift to expensive procedures postacquisition is also clearly an important question.

The price differences across all studies are due to diverse PE firm strategies and their ability to roll up practices in particular markets. The studies generally did not examine quality of care, so it is unclear if quality improved, particularly for the studies finding the largest price increases. The one exception was the neonatology study, which had the highest price increase, but the acquisitions were not associated with improvements in readmission rates or patient outcomes.^9^

The health care spending increase of 16.0% that we found was generally less than what other studies found (when measured), mostly because expenditure increases are directly related to price increases. The low price increases found in dermatology did not lead to significant expenditure increases,^8^ whereas the high price increases found in gastroenterology (34.7%) and neonatology (70.4%) contributed to large expenditure increases of 48.9% and 56.4%, respectively.^7,9^

The results of our economic evaluation have answered some questions and left others open. The inclusion of physician fixed effects in our models made it clear that it was individual gastroenterologists who were performing more colonoscopies after PE acquisition (at least for enrollees of the insurers in our data); it was not just the case that their practice did more colonoscopies, which could happen simply through hiring more gastroenterologists rather than increasing the workload of current physicians at the practice. How gastroenterologists could perform more colonoscopies is beyond the scope of this study, but one possibility is that advanced practice providers (eg, nurse practitioners, physician assistants) screened new consultations, leaving physicians with more time for procedures.^18^ The more colonoscopies per physician result helped hone in on the types of strategies PE firms may be using to increase utilization and, in turn, revenue.

### Limitations

This study had several limitations. First, despite efforts to use and validate comprehensive data on PE firms acquiring physician practices, observed acquisitions were limited to those publicly announced, which can lead to underreporting of small acquisitions, potentially biasing our results toward a null result. Second, our power to detect changes for rare events for some of our quality measures (eg, perforation, infection) may be limited. To address this, we analyzed complications in broader categories rather than individually. Third, one of the assumptions of our model is that the regressors are not correlated with the error terms. This assumption could be violated if the errors are correlated with unobserved, time-varying characteristics. Our inclusion of fixed effects and patient characteristics is to help avoid this possibility. However, selection and changes in physician behavior could still pose a concern for this assumption. Physicians’ decisions to sell to PE are unlikely to be exogenous. As an example, physicians struggling financially, perhaps due to a greater proportion of patients with low income or at higher risk for complications, may be more likely to sell to PE. The inclusion of patient characteristics in our model attempted to alleviate this concern, but it was not a concern we could fully eliminate.

## Conclusions

This economic evaluation showed PE acquisition of gastroenterology practices was associated with increases in price, spending, and utilization, but was not associated with changes in quality. This study adds to the literature on price, spending, and utilization increases stemming from PE acquisition and is one of the first to simultaneously analyze quality and show a larger price effect for PE practices with more market share. Our study adds to the evidence base of the need for transparency about corporate influence in health care, particularly PE firms.

## Notes

Supplement 1. **eTable 1. **List of codes used to calculate quality measures **eMethods. **Empirical strategy details **eTable 2. **PE acquisitions of gastroenterology practices by year **eTable 3. **Number of practice sites and physicians in sample by year **eTable 4. **Number of years of data each practice site contributes to the analytic sample **eFigure 1. **Raw trends by treatment status **eFigure 2. **Price event study for high market share treated colonoscopies **eTable 5. **Summary of DID estimates -- 2012-2019 study period **eFigure 3. **Price event study after excluding 2015 acquisitions

Supplement 2. Data sharing statement
