# Supplementary material for: Private Equity Acquisition of Gastroenterology Practices and Colonoscopy Price and Quality
Source: JAMA Health Forum. 2025 Jun 20;6(6):e251476. doi: 10.1001/jamahealthforum.2025.1476 (PMC12181784; doi:10.1001/jamahealthforum.2025.1476)
Supplement: Supplement 1. — eTable 1. List of codes used to calculate quality measures eMethods. Empirical strategy details eTable 2. PE acquisitions of gastroenterology practices by year eTable 3. Number of practice sites and physicians in sample by year eTable 4. Number of years of data each practice site contributes to the analytic sample eFigure 1. Raw trends by treatment status eFigure 2. Price event study for high market share treated colonoscopies eTable 5. Summary of DID estimates -- 2012-2019 study period eFigure 3. Price event study after excluding 2015 acquisitions [file jamahealthforum-e251476-s001.pdf]

Supplemental Online Content

Arnold DR, Fulton BD, Abdelhadi OA, Teotia A, Scheffler RM. Private equity acquisition of gastroenterology practices and colonoscopy price and quality. *JAMA Health Forum.* 2025;6(6):e251476. doi:10.1001/jamahealthforum.2025.1476

eTable 1. List of codes used to calculate quality measures

eMethods. Empirical strategy details

eTable 2. PE acquisitions of gastroenterology practices by year

eTable 3. Number of practice sites and physicians in sample by year

eTable 4. Number of years of data each practice site contributes to the analytic sample

eFigure 1. Raw trends by treatment status

eFigure 2. Price event study for high market share treated colonoscopies

eTable 5. Summary of DID estimates -- 2012-2019 study period

eFigure 3. Price event study after excluding 2015 acquisitions

This supplemental material has been provided by the authors to give readers additional information about their work.

© 2025 Arnold DR et al. *JAMA Health Forum*.

eTable 1. List of codes used to calculate quality measures

Quality Measure	Code type	Codes
Colonoscopy	HCPCS	44388, 44389, 44391, 44392, 44393, 44394, 44397, 45378, 45379, 45380, 45381, 45382, 45383, 45384, 45385, 45386, 45391, 45392, G0105, G0121
Polypectomy	HCPCS	44389, 45380, 44392, 45384, 44393, 44394, 45383, 45385, 45379
Incomplete colonoscopy	CPT modifier	53, 73, 74
Cardiac complications	ICD-9/ICD-10	Arrhythmia (ICD-9 codes: 427.0-427.4, 427.6-427.9; ICD-10 codes: I47.1, I47.2, I47.9, I49.9)<br>Congestive heart failure (ICD-9 codes: 428.0-428.9; ICD-10 codes: I50.1, I50.810-I50.814, I50.82-I50.84, I50.89, I50.9)<br>Cardiac or respiratory arrest (ICD-9 codes: 427.5, 799.1, 997.1; ICD-10 codes: I46.9, I97.710, I97.790, I97.88, I97.89, R09.2)<br>Syncope, hypotension, or shock (ICD-9 codes: 453.29, 458.8-458.9, 639.5, 780.2, 785.50-785.51, 998.0, 995.4; ICD-10 codes: I95.89, I95.9, O08.3, R55, R57.0, R57.9, I88.2XXA)
Serious GI complications	ICD-9/ICD-10	Perforation (ICD-9 codes: 569.83, 998.2; ICD-10 codes: K63.1, K91.71, K91.72)<br>Lower gastrointestinal bleeding (ICD-9 codes: 558.9, 578.1, 995.2, 995.89, 998.1-998.13, 286.5, 459, 562.02-562.03, 562.12, 562.13, 569.3, 569.84-569.86, 578.9, 792.1; ICD-10 codes: K52.3, K52.89, K52.9, K92.1, T88.51XA, K91.61, K91.62, K91.840, K91.841, K91.870, K91.871, K91.872, K91.873, K57.11, K57.13, K57.31, K57.33, K62.5, K55.20, K55.21, K63.81, K92.2, R19.5)<br>Infection (CPT code 78066; ICD-9 codes: 790.7, 424.9-424.99; ICD-10 codes: R7881, I39, I38)
Other GI complications	ICD-9/ICD-10	Paralytic ileus (ICD-9 code: 560.1; ICD-10 codes: K56.0, K56.7)<br>Nausea, vomiting, dehydration (ICD-9 codes: 276.5, 536.2, 787.0-02; ICD-10 codes: R11.10, R11.2, R11.0)<br>Abdominal pain (ICD-9 code: 789.0)<br>Diverticulitis (ICD-9 codes: 562.01, 562.03, 562.11, 562.13; ICD-10 codes: K57.12, K57.13, K57.32, K57.33)<br>Enterocolitis (ICD-9 codes: 555-556; ICD-10 codes: K50.00, K50.10, K50.80, K50.90, K51.80)

Abbreviations: HCPCS = Healthcare Common Procedure Coding System, CPT = Current Procedural Terminology, ICD = International Classification of Diseases

© 2025 Arnold DR et al. JAMA Health Forum.

eMethods. Empirical strategy details

Our empirical strategy is based on a difference-in-differences (DID) event study with physician and year fixed effects. Under certain assumptions that we describe in the next paragraph, the coefficient for the treatment variables in our model can provide a causal interpretation of how a private equity acquisition affects healthcare prices and quality. The unit of analysis in our model is a colonoscopy and we use various patient characteristics as controls. The treatment variable in our setting is based on the PE ownership variable of the physician who performs the procedure. This allows multiple colonoscopies performed on the same patient to have different treatment status if the patient received multiple colonoscopies from different physicians. The majority of patients (97%), however, received only one colonoscopy during our study period. We used the following model to perform our DID event study analysis:

$$Y_{ijt} = \sum_{p=-5}^{5} (\lambda_p PE_i \times post_{ip}) + \beta X_{ijt} + \gamma_i + \tau_t + \epsilon_{ijt}$$

where Y represents outcome variables such as price, spending, utilization, or various measures of quality, PE is a binary variable that indicates whether the practice of the physician performing the colonoscopy was acquired by PE during the study period, $post_{ip}$ is a vector of variables in which p is the number of years from the the year that the physician's practice was acquired by PE (e.g., for a practice that was acquired by PE in 2017, then p=-3 in 2014, p=-2 in 2015, p=-1 in 2016, p=0 in 2017, …, p=4 in 2021), X is a vector of patients characteristics including age (using six age bands), sex, and diagnosis (using ICD 9/10 codes), γ is a physician fixed effect, τ is a year fixed effect, and ϵ is an error term. In the context of heterogeneous treatment effects and staggered interventions (our context), a recent econometric literature has shown that the coefficients arising from ordinary least squares (OLS) two-way fixed effect events can be

© 2025 Arnold DR et al. JAMA Health Forum.

biased.[1,2] To avoid this bias, all the event studies we present are based on the Sun & Abraham (2021) model that produces unbiased event study estimates.[3]

References

1. de Chaisemartin C, D'Haultfœuille X. Two-way fixed effects and differences-in-differences with heterogeneous treatment effects: a survey. Econom J. 2023;26(3):C1-C30. doi:10.1093/ectj/utac017
2. Roth J, Sant'Anna PHC, Bilinski A, Poe J. What's trending in difference-in-differences? A synthesis of the recent econometrics literature. J Econom. 2023;235(2):2218-2244. doi:10.1016/j.jeconom.2023.03.008
3. Sun L, Abraham S. Estimating dynamic treatment effects in event studies with heterogeneous treatment effects. J Econom. 2021;225(2):175-199. doi:10.1016/j.jeconom.2020.09.006

© 2025 Arnold DR et al. JAMA Health Forum.

eTable 2. PE acquisitions of gastroenterology practices by year

	Treated	
Year	Number of practice sites	Number of physicians
2015	354	351
2016	112	156
2017	104	80
2018	194	247
2019	154	201
2020	137	232
2021	184	226
TOTAL	1,240	1,494

Source: Authors' analysis of data from Health Care Cost Institute (HCCI), Pitchbook, Irving Levin Associates, and IQVIA's OneKey.

© 2025 Arnold DR et al. JAMA Health Forum.

eTable 3. Number of practice sites and physicians in sample by year

	Treated		Control	
	Number of practice sites	Number of physicians	Number of practice sites	Number of physicians
2012	490	1,043	1450	1,733
2013	505	1,111	1457	1,747
2014	526	1,147	1385	1,672
2015	541	1,182	1305	1,574
2016	554	1,234	1283	1,543
2017	574	1,286	1257	1,520
2018	624	1,303	1262	1,533
2019	586	1,302	1228	1,501
2020	549	1,326	1004	1,324
2021	492	1,354	913	1,246
Unique over study period	1,240	1,494	2,657	2,550

Source: Authors' analysis of data from Health Care Cost Institute (HCCI), Pitchbook, Irving Levin Associates, and IQVIA's OneKey.

© 2025 Arnold DR et al. JAMA Health Forum.

eTable 4. Number of years of data each practice site contributes to the analytic sample

	Treated	Control
1	301	510
2	200	373
3	103	221
4	85	188
5	81	154
6	71	174
7	61	157
8	88	223
9	69	139
10	181	518
Unique practice sites over study period	1,240	2,657

Source: Authors' analysis of data from Health Care Cost Institute (HCCI), Pitchbook, Irving Levin Associates, and IQVIA's OneKey.

© 2025 Arnold DR et al. JAMA Health Forum.

eFigure 1. Raw trends by treatment status

Panel A: Price

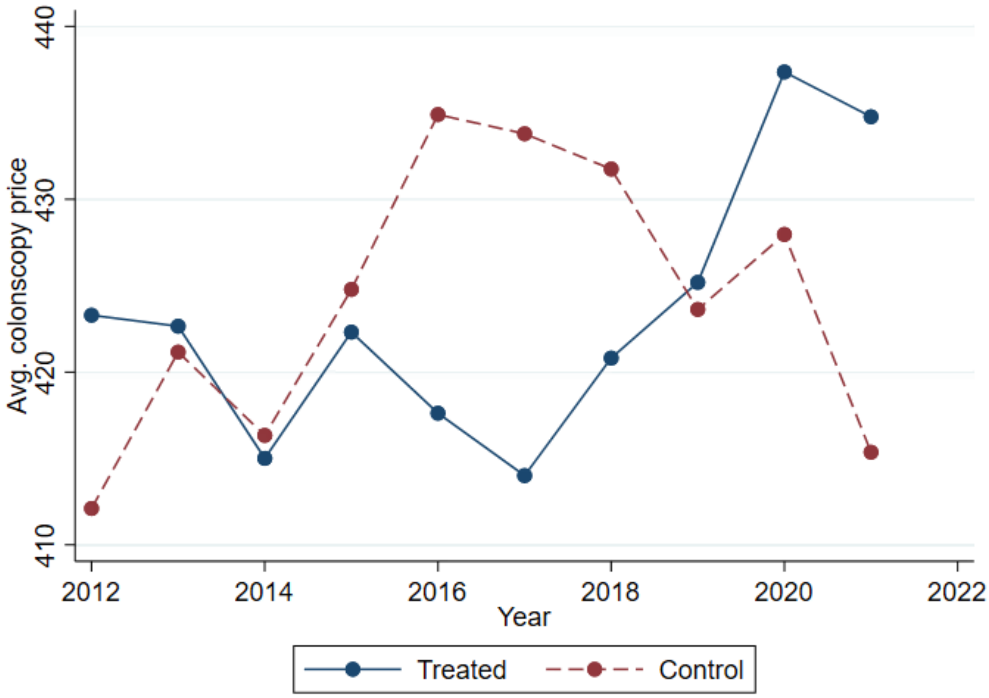

Panel B: Spending

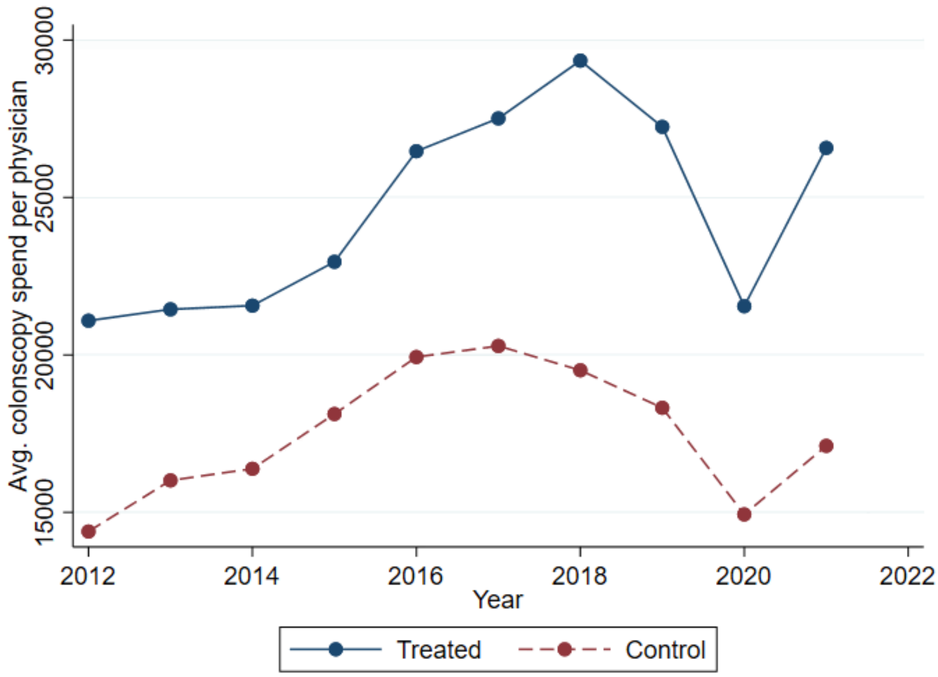

© 2025 Arnold DR et al. JAMA Health Forum.

Panel C: Utilization

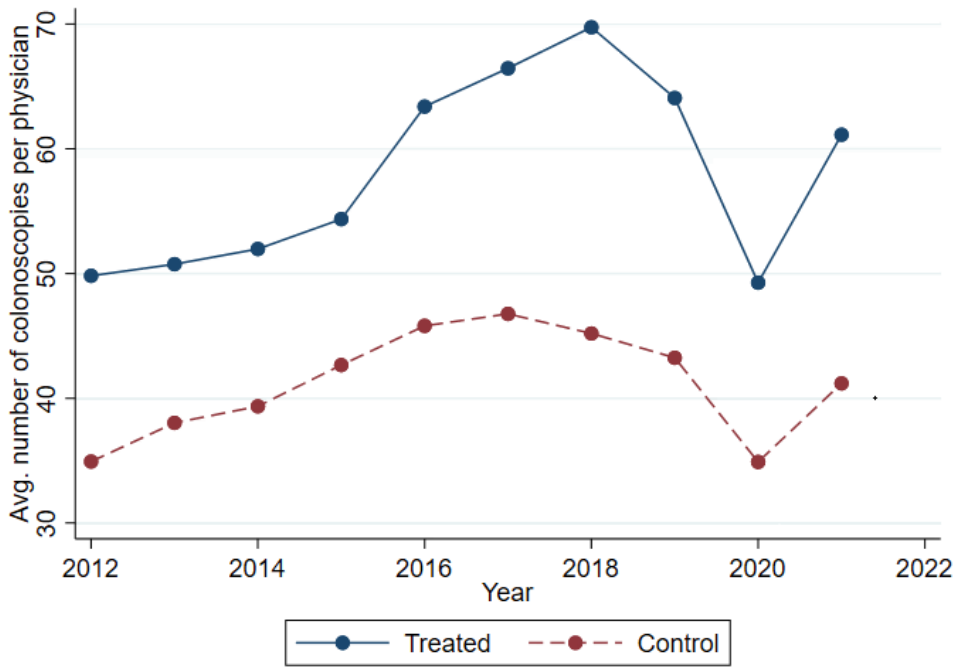

Panel D: Patients per physician

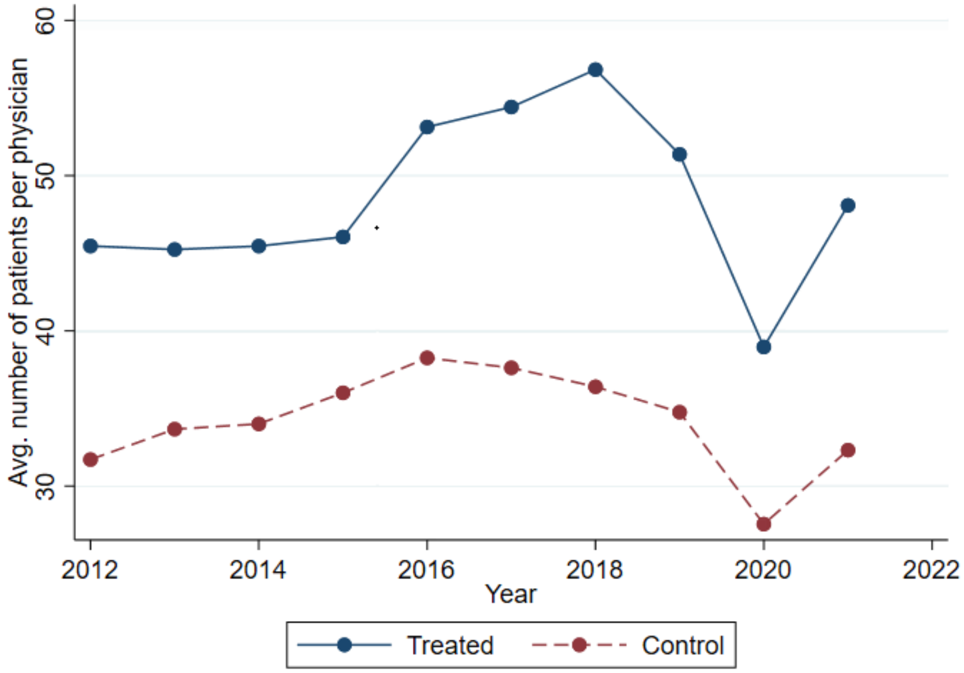

Source: Authors' analysis of data from Health Care Cost Institute (HCCI), Pitchbook, Irving Levin Associates, and IQVIA's OneKey.

© 2025 Arnold DR et al. JAMA Health Forum.

eFigure 2. Price event study for high market share treated colonoscopies

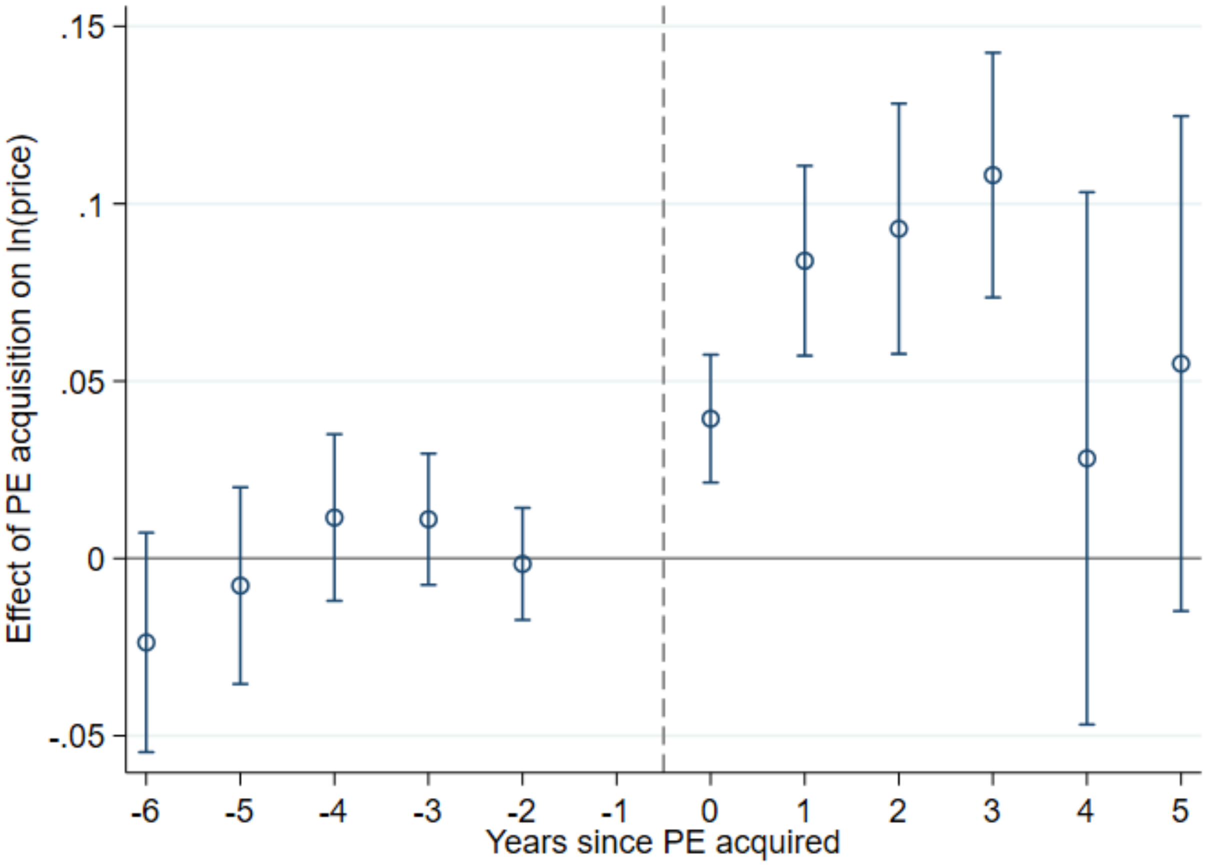

Source: Authors' analysis of data from Health Care Cost Institute (HCCI), Pitchbook, Irving Levin Associates, and IQVIA's OneKey.

Notes: Only colonoscopies performed by gastroenterologists associated with PE practices above the market share 75th percentile (24.4%) are considered treated. Sun & Abraham (2021) event study coefficients. The unit of analysis was a colonoscopy. All regressions included physician and year fixed effects as well as controls for patient age (measured in age bands), sex, and CPT code. Standard errors are clustered at the practice site level.

© 2025 Arnold DR et al. JAMA Health Forum.

eTable 5. Summary of DID estimates -- 2012-2019 study period

	DID estimate [95% CI]
ln(price)	**2.9% [0.9% - 5.0%]**
ln(colonoscopy spending per physician)	**11.1% [3.6% - 19.2%]**
ln(colonoscopies per physician)	**9.2% [2.2% - 16.7%]**
ln(colonoscopy patients seen per physician)	**8.5% [1.5% - 16.1%]**
Quality Measures	
Polypectomy	0.0081065 [-0.0000875 - 0.0163006]
Incomplete colonoscopy	0.0002981 [-0.000343 - 0.0009392]
Cardiovascular complications	-0.0000717 [-0.0007863 - 0.000643]
Serious GI	-0.0015746 [-0.003670 - 0.005212]
Nonserious GI	0.00046 [-0.001176 - 0.002096]
Any complication	-0.001095 [-0.0038468 - 0.0016567]

Abbreviations: ln, natural log; PE, private equity.
Bold indicates the Sun & Abraham (2021) difference-in-differences (DID) coefficient is statistically significant at the $p<0.05$ level. The estimated DID coefficients associated with the natural log dependent variables were converted to the percentages shown in the table using the formula (exp(coef)-1)*100. The unit of analysis was a colonoscopy. Each process and post-procedure complication measure is a 0/1 variable. All regressions included physician and year fixed effects as well as controls for patient age (measured in age bands), sex, and CPT code. Standard errors are clustered at the practice site level.

© 2025 Arnold DR et al. JAMA Health Forum.

eFigure 3. Price event study after excluding 2015 acquisitions

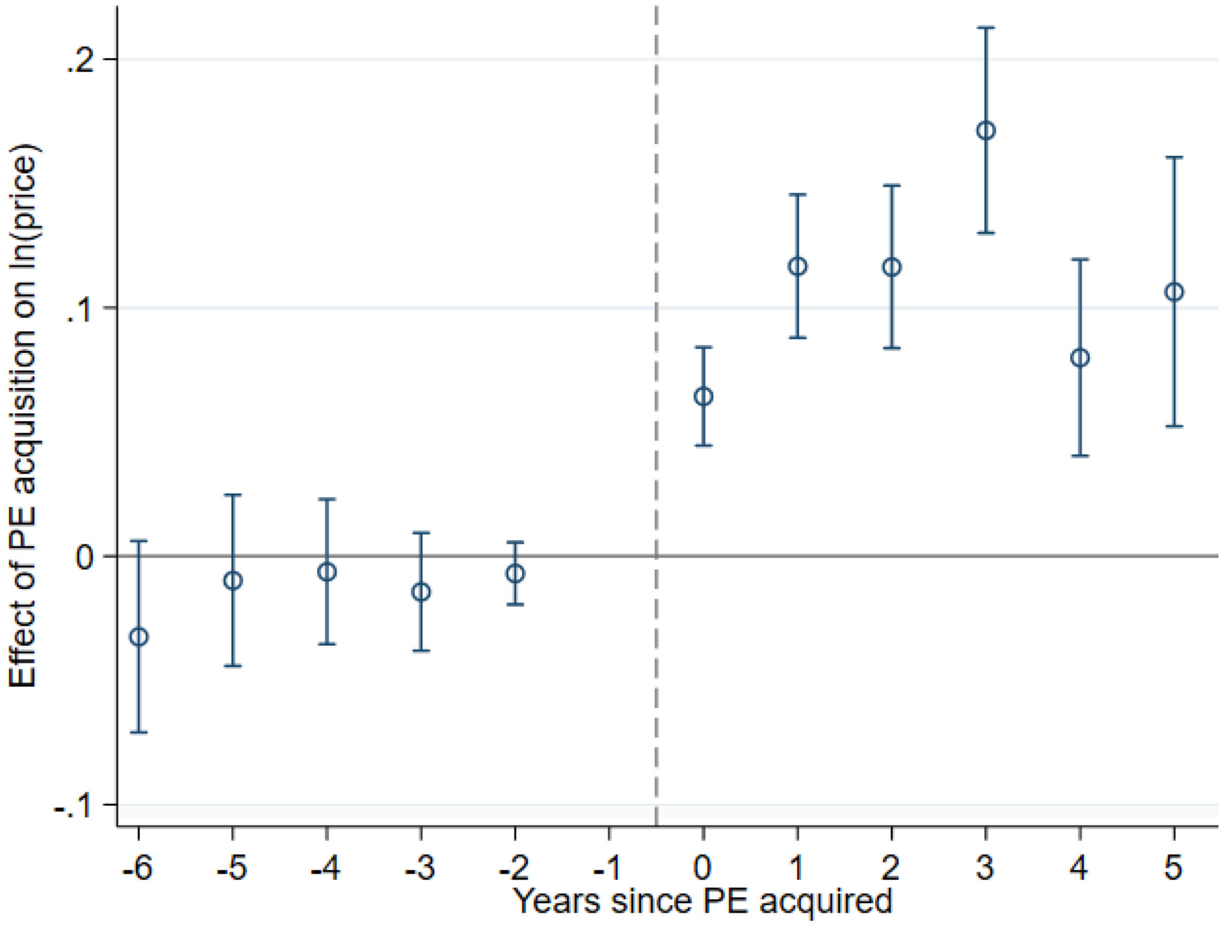

Source: Authors' analysis of data from Health Care Cost Institute (HCCI), Pitchbook, Irving Levin Associates, and IQVIA's OneKey.

Notes: Only colonoscopies performed by gastroenterologists associated with PE practices above the market share 75th percentile (24.4%) are considered treated. Sun & Abraham (2021) event study coefficients. The unit of analysis was a colonoscopy. All regressions included physician and year fixed effects as well as controls for patient age (measured in age bands), sex, and CPT code. Standard errors are clustered at the practice site level.

© 2025 Arnold DR et al. JAMA Health Forum.
