# Supplementary material for: Private Equity Acquisition of Gastroenterology Practices and Colonoscopy Price and Quality
Source: JAMA Health Forum. 2025 Jun 20;6(6):e251476. doi: 10.1001/jamahealthforum.2025.1476 (PMC12181784; doi:10.1001/jamahealthforum.2025.1476)
Supplement: Supplement 2. — Data sharing statement [file jamahealthforum-e251476-s002.pdf]

Data Sharing Statement

Arnold. Private Equity Acquisition of Gastroenterology Practices and Colonoscopy Price and Quality. *JAMA Health Forum*. Published June 20, 2025.
doi:10.1001/jamahealthforum.2025.1476

Data
Data available: No

Additional Information
Explanation for why data not available: Our DUA precludes sharing of the data.
